# Outcomes of an inpatient refeeding protocol in youth with anorexia nervosa: Rady Children’s Hospital San Diego/University of California, San Diego

**DOI:** 10.1186/s40337-016-0132-0

**Published:** 2017-01-03

**Authors:** Tamara R. Maginot, Maya M. Kumar, Jacqueline Shiels, Walter Kaye, Kyung E. Rhee

**Affiliations:** 1 Department of Psychiatry, University of California, San Diego School of Medicine, 4510 Executive Drive, Suite 315, San Diego, CA 92121 USA; 2 Division of Academic General Pediatrics and Community Health, Department of Pediatrics, University of California, San Diego School of Medicine and Rady Children’s Hospital of San Diego, 7910 Frost Street, Suite 300, San Diego, CA 92123 USA; 3 9500 Gilman Drive, MC 0874, La Jolla, CA 92093 USA

**Keywords:** Nutritional rehabilitation, Refeeding syndrome, Hypophosphatemia, Hypomagnesemia, Hypokalemia, Medical stabilization, Eating disorders, Anorexia nervosa, Avoidant Restrictive Food Intake Disorder

## Abstract

**Background:**

Current guidelines for nutritional rehabilitation in hospitalized restrictive eating disorder patients recommend a cautious approach to refeeding. Several studies suggest that higher calorie diets may be safe and effective, but have traditionally excluded severely malnourished patients. The goal of this study was to evaluate the safety of a higher calorie nutritional rehabilitation protocol (NRP) in a broad sample of inpatients with restrictive eating disorders, including those who were severely malnourished.

**Methods:**

A retrospective chart review was conducted among eating disorder inpatients between January 2015 and March 2016. Patients were started on a lower calorie diet (≤1500 kcals/day) or higher calorie diet (≥1500 kcals/day). Calorie prescription on admission was based on physician clinical judgement. The sample included patients aged 8–20 years with any DSM-5 restrictive eating disorder. Those who were severely malnourished (<75% expected body weight [EBW]) or required tube feeding during admission were included. Multivariable regression models were used to determine whether level of nutritional rehabilitation was associated with hypophosphatemia, hypomagnesemia, or hypokalemia.

**Results:**

The sample included 87 patients; mean age was 14.4 years (S.D. 32.7); 29% were <75% EBW. The majority (75.8%) was started on higher calorie diets (mean 1781 kcal/day). Controlling for rate of calorie change, initial %EBW, age, race/ethnicity, insurance, diagnosis, and NG/NJ tube placement, higher calorie diets were not associated with hypophosphatemia, hypomagnesemia, or hypokalemia on admission or within the first 72 h. Increased risk of hypophosphatemia on admission was associated with lower baseline %EBW.

**Conclusion:**

A higher calorie NRP was tolerated in this broad population of inpatients with restrictive eating disorders. Lower %EBW on admission was a more important predictor of hypophosphatemia than initial calorie level. Larger studies are required to demonstrate the safety of higher calorie diets in severely malnourished patients.

## Plain english summary

Refeeding syndrome is a concern for patients with eating disorders who are undergoing nutritional rehabilitation. It is traditionally recommended to start at a lower calorie level and advance slowly as the safest means of treatment. However, in this sample of 87 patients with restrictive eating disorders hospitalized with medical complications of malnutrition at Rady Children’s Hospital, San Diego/University of California San Diego, starting at a higher calorie level was not associated with an increased risk of electrolyte abnormalities. However, patients presenting at <75% of expected body weight were at increased risk of developing hypophosphatemia regardless of initial calorie level. Further research is needed to determine if this method of calorie advancement is truly safe in patients with severe malnutrition (i.e., those <75% of expected body weight).

## Background

In managing patients with moderate to severe malnutrition secondary to restrictive eating disorders, the optimal rate of nutritional rehabilitation remains a subject of debate. Guidelines by the American Psychiatric Association [1] and Academy of Nutrition and Dietetics [2] currently recommend a conservative approach to nutritional rehabilitation to prevent refeeding syndrome. Refeeding syndrome occurs when malnourished patients transition to using dietary carbohydrates rather than stored macronutrients as their primary source of energy [3–5]. As the body shifts from a chronically catabolic state to an anabolic state, low body stores of phosphorus, magnesium, and potassium, in conjunction with intracellular shifting of these electrolytes (a consequence of an exaggerated insulin response) leads to low serum electrolyte levels [3–5]. Clinical sequelae may be severe, including muscle weakness and cramping, cardiac arrhythmias, vomiting, seizures, delirium, and death [3–6]. Low calorie diets have traditionally been recommended to prevent this complication during weight restoration [1, 2]. However, this approach to refeeding increases the risk of providing inadequate nutrition to cover baseline energy expenditure and facilitate weight restoration [7, 8]. This may result in slow weight gain or even weight loss during the refeeding process, leading to prolonged medical complications related to malnutrition (e.g., bradycardia) and longer hospitalizations [7–10].

Recent research has examined whether a less conservative approach to nutritional rehabilitation can be safely performed, particularly in those with restrictive eating disorders [7–19]. Several groups have evaluated the effect of starting on a higher calorie diet (ranging from 1400 to 2400 kcals) among patients aged 10 to 21 years old and report shorter hospital stays [11, 13], faster weight gain [12–14], a low rate of developing hypophosphatemia during nutritional rehabilitation, and no incidence of clinical refeeding syndrome [8–15]. While rate of calorie advancement varied among groups, increasing 250 kcals on day 2 and 3, and then by 250 kcals every other day until day 7, was not associated with increased risk of electrolyte abnormalities and resulted in faster weight gain [14]. Many of these groups started patients on prophylactic phosphorus supplementation which may have helped prevent the development of electrolyte abnormalities or clinical refeeding syndrome [10, 12, 13]. The use of NG tubes for nutritional rehabilitation was also not associated with adverse outcomes [12, 16, 17]. Overall, these studies suggest that starting at a higher calorie level and advancing quickly may be considered in moderately malnourished (75–85% of expected body weight) adolescents and young adults with AN.

Unfortunately, there is limited literature on the safety of refeeding at higher calorie levels in severely malnourished patients with eating disorders. Golden et al. [11] examined a subgroup of 49 severely malnourished adolescents with anorexia nervosa (age 11–16 years) presenting at <70% mBMI. Within this group, prescribed calorie level did not change the risk of electrolyte abnormalities associated with refeeding syndrome [11]. Studies have concluded that percent body weight on admissions was more associated with the development of hypophosphatemia than initial calorie prescription [9–11]. Two small descriptive studies examining patients presenting with a BMI < 12 (ranging in age from 11 to >40 years old) found that these patients could be safely refed with <1200 kcal per day, but did not assess the safety of a higher calorie diet [16, 17]. A recent systematic review concluded that there is currently insufficient evidence to support using higher calorie prescriptions in severely malnourished patients and more research is needed in this area to ascertain the safety of starting on a higher calorie diet [18].

The primary goal of this study is to describe the nutritional rehabilitation protocol implemented at the University of California, San Diego (UCSD)/Rady Children’s Hospital San Diego (RCHSD) Medical Behavioral Unit (MBU), and to examine whether higher calorie nutritional rehabilitation protocols (NRP) increase the risk of electrolyte abnormalities associated with refeeding syndrome among patients with restrictive eating disorders. The secondary goal of this study is to examine the risk of a higher initial calorie prescription in the subgroup of patients who were severely malnourished at presentation. This study builds upon existing literature by including a heterogeneous population of patients with restrictive eating disorders, including those with Avoidant Restrictive Food Intake Disorder (ARFID) or Other Specified Eating Disorder as defined in the Diagnostic and Statistical Manual, fifth edition (DSM 5) [19] and those who require NG tube feeding.

## Methods

### Study design and subjects

This is an observational study of current practices on the Medical Behavioral Unit (MBU) at Rady Children’s Hospital San Diego (RCHSD)/UCSD. A retrospective chart review was conducted among patients admitted between January 2015 and March 2016. RCHSD is the only children’s hospital in San Diego County, serving several neighboring counties in Southern California. The MBU is an inpatient unit at RCHSD with 10 medical-surgical beds for patients with medical complications of malnutrition.

Patients were included if they were diagnosed with AN, Other Specified Eating Disorder or Avoidant Restrictive Food Intake Disorder (ARFID) based on the *Diagnostic and Statistical Manual of Mental Disorders, Fifth Edition* (DSM-5) [19], and met medical criteria for hospitalization. Hospitalization criteria were based on those recommended by the Society for Adolescent Health and Medicine [8] and include: weight <75% of the expected body weight (EBW); resting heart rate (HR) < 45 beats per minute (bpm) while asleep or <50 bpm while awake; hypotension (<90/45 mmHg); body temperature < 35.6° Celsius; orthostatic change in systolic blood pressure (SBP) > 20 mmHg or diastolic blood pressure (DBP) > 10 mmHg; orthostatic change in HR > 30 bpm; syncope, arrhythmia, heart failure, or symptomatic pericardial effusion related to malnutrition or refeeding; dehydration; electrolyte disturbance related to malnutrition, refeeding, vomiting, or laxative/diuretic abuse; or acute food refusal necessitating tube-feeding or non-enteric feeding.

Patients were between the ages of 8 and 20 years old and were included in the analysis if this was their first or second admission to the (MBU), with no previous admission to the MBU in the preceding 30 days. Patients were excluded if they had a diagnosis of bulimia nervosa (*n* = 1), left the hospital against medical advice (*n* = 3), or were transferred to another medical or psychiatric facility (*n* = 2). Human subjects approval was obtained from the UCSD Human Research Protections Program Institutional Review Board.

### Nutritional rehabilitation protocol

Upon admission, patients were started on an oral nutritional rehabilitation diet; the admitting physician relied on clinical judgment to determine the initial calorie level based on the patient’s recent dietary history. Initial caloric level typically ranged from 1500 to 1800 kcal/day, but lower calorie diets (typically 1200 kcal/day) were used if extreme dietary restriction was reported (e.g., <500 kcal/day for several weeks). Daily caloric intake was titrated to achieve 150–300 g of weight gain per day, and an overall goal of 1–2 kg of weight gain per week. If the daily expectation for weight gain was not met for 2 days in a row, caloric intake was increased. Caloric intake was also advanced if the patient had persistent or severe cardiac complications (e.g., heart rate <40 bpm overnight, symptomatic postural changes in HR or BP, arrhythmia) despite meeting daily weight restoration goals. When increased, daily caloric intake was advanced in increments of 300 kcal/day.

Nutritional rehabilitation was provided orally as three meals per day and up to three snacks per day. The MBU oral diet consisted of a 7-day rotating menu. Macronutrient content of the diet was approximately 31–36% fat, 14–19% protein, and 50% carbohydrate. All patients were required to eat in a group setting, supervised by a licensed psychiatry technician or vocational nurse, and were observed for at least 30 min after each meal or snack. Patients who refused to eat the provided food were offered the caloric equivalent as a liquid nutritional supplement (Boost™ or Nutren™, either the 1.0 kcal/mL or 1.5 kcal/mL concentration), which was comparable in macronutrient composition to the oral diet.

If the patient had difficulty eating or taking liquid supplement by mouth within the specified time limit, a nasogastric (NG) or nasojejunal (NJ) tube was inserted to provide nutrition. The NG/NJ tube was left in place and used to supplement oral intake until the patient consumed 100% of daily nutritional and fluid requirements by mouth for at least 24 h. Decisions about calorie advancement for patients receiving tube feeds were made using identical criteria as for those who were consuming all nutrition orally (see above). Therefore, patients receiving some NG/NJ feeds during their admission were included in this analysis.

Intravenous fluids were only used in dehydrated patients who could not tolerate oral fluid replacement. A multivitamin was routinely recommended by the dietitian and additional micronutrient supplements (e.g., Vitamin D, Vitamin B12, zinc, or folate) were provided as needed.

Refeeding labs were monitored at least once daily. Patients did not receive routine prophylaxis against refeeding syndrome. Electrolyte supplementation was only initiated when decreases in serum electrolytes were identified. There was no standard protocol defining when patients should be started on electrolyte supplementation; the attending physician used clinical judgment and often started electrolyte supplementation prior to serum levels falling into the abnormal range if levels were noted to be dropping rapidly.

All patients were routinely assessed and followed by psychologists, who provided family, group, and individual therapy. Psychiatry assessments were requested based on the patient’s clinical condition.

### Measures

On admission, all patients were screened with a complete blood count and differential, complete metabolic panel, serum magnesium, serum phosphorus, prealbumin, thyroid function tests, serum zinc, serum Vitamin D-25-OH, C-reactive protein, erythrocyte sedimentation rate, serum estradiol or testosterone, urinalysis, and electrocardiogram. Subsequently, daily refeeding labs were assessed including a basic metabolic panel, serum magnesium, serum phosphorus, and urinalysis. Lowest overnight HR was obtained from continuous cardiac monitoring overnight, performed for all patients throughout their stay. Orthostatic BP and HR were obtained first thing in the morning throughout the admission. Weights were also obtained daily throughout the admission after the first morning void, wearing only a hospital gown. Height was obtained on day 1 of admission.

Overall change in calories was determined by subtracting the initial calorie level from the discharge calorie level. The rate of change in calories was calculated as overall change in calories divided by the total length of hospital stay in days. Change in lowest overnight HR was also calculated in a similar manner (lowest overnight HR the night prior to discharge – lowest overnight HR in the first 48 h of admission).

Expected body weight (EBW) was determined using several clinical factors. While some groups conventionally use the 50th percentile BMI to calculate expected body weight for children and adolescents [20], our program believes in customizing EBW to return each child or adolescent to the growth trajectory in which they were previously healthy. If a premorbid growth curve was available, EBW was selected to restore the patient back to the patient’s premorbid BMI percentile (25th, 50th, or 75th percentile BMI). If the patient’s premorbid BMI was >85th percentile but the patient was otherwise healthy, initial EBW was selected to restore the patient to a BMI at the 75th percentile. If premorbid growth patterns were unknown, the 50th percentile BMI for age and sex was used to determine EBW. Since height was only measured on day 1, changes in BMI during the hospital stay essentially reflected changes in weight. Percent of expected body weight (%EBW) was calculated as weight divided by EBW. Change in %EBW was determined by subtracting the admission %EBW from the discharge %EBW. Rate of change in %EBW was calculated as the overall change in %EBW divided by the total length of stay. Severe malnutrition was defined as a ≤ 75% EBW and moderate malnutrition was defined at 75–85% EBW.

Electrolyte abnormalities were defined as a low phosphorus, magnesium, or potassium within the first 72 h of admission. In the current analysis, hypophosphatemia was defined as serum phosphorus <3.0 mg/dL, hypomagnesemia was defined as serum magnesium <1.7 mg/dL, and hypokalemia was defined as serum potassium <3.3 mmol/L [11].

### Analysis

Means and frequencies, *t*-tests, and chi-square tests were used to describe the sample and compare those who were started on a lower calorie nutritional rehabilitation diet (≤1500 kcals/day) to those who were started on a higher calorie diet (≥1500 kcals/day). The aim of this study was to examine whether starting on a higher or lower calorie nutritional rehabilitation diet affected overall change in %EBW, overall change in calories, rate of change in %EBW, rate of change in calories, change in HR, change in weight, length of hospital stay (LOS), and electrolyte abnormalities (specifically hypophosphatemia, hypomagnesemia, or hypokalemia). Multivariable general linear regression models were used to examine the relationship between overall change in %EBW and calories, rate of change in %EBW and calories, change in HR and weight, LOS, and level of initial nutritional rehabilitation (high vs. low). %EBW on admission was included as a covariate in all models since this indicated severity of illness and may have been used by the physician to determine what calorie level to initiate. Standard demographic characteristics, diagnosis, and covariates with a *p*-value ≤0.05 were also included in the models. Significance level was set at *p* ≤ 0.05. Multivariable logistic regression models were used to determine whether level of nutritional rehabilitation was associated with hypophosphatemia, hypomagnesemia, or hypokalemia on the first day of admission and within the next 72 h. %EBW, rate of change in calories, standard demographic characteristics, diagnosis, and covariates with a *p*-value ≤0.05 were included as covariates. SAS v9.4 (Cary, NC) was used for analysis.

## Results

A total of 87 patients hospitalized during the study period met inclusion criteria. The mean age of patients was 14.4 years (S.D. 2.7 years) with a range of 8 to 20 years. The mean LOS was 15.3 days (S.D. 9.6 days). Patients had a %EBW of 80.6% (S.D. 10.5%) on admission and gained an average of 3.1 kg (S.D. 2.1 kg) during their stay. This translates to a mean change in %EBW of 5.41% (S.D. 4.32%) during their stay, or 0.35%EBW/day (S.D. 0.30% EBW/day). Most patients had AN-restrictive subtype (66.7%); 16.1% had AN-binge/purge subtype, 5.7% were diagnosed with Other Specified Eating Disorder, and 11.5% were diagnosed with ARFID. Fifteen patients (13.8%) required nasogastric/nasojejunal (NG/NJ) feeds during admission. The overall rate of change in calories was 109 kcals/day (S.D. 50.1 kcal/day).

Baseline characteristics of patients in the low and high calorie groups are summarized in Table 1. The majority (75.8%) of patients were started on a higher calorie diet on admission (mean 1781 kcal/day, S.D. 315 kcal/day, range 1500–3000 kcal/day). The low calorie group received a mean of 1185 kcal/day (S.D. 65 kcal/day, range 1000–1300 kcal/day). Patients in the low calorie group had longer LOS (20.7 days vs. 13.5 days, *p* < 0.01). They were also more likely to have an NG/NJ tube placed (28.6% vs. 9.1%, *p* = 0.02). There was no difference in diagnosis by calorie level. There were no differences in mean laboratory values except that patients in the low calorie group had higher ALT (38.3 vs. 31.9, *p* = 0.05) (Table 2). The low calorie group also had higher overnight HR on admission (49.4 vs 43.8, *p* = 0.05). There were no differences in orthostatic vital signs or ECG findings.

**Table 1:** Characteristics of patients admitted to the Medical Behavioral Unit (*n* = 87)

	Low Calorie (*n* = 21)			High Calorie (*n* = 66)			
	Mean (S.D.)	Min	Max	Mean (S.D.)	Min	Max	*P*-value
Age (yrs)	14.66 (2.83)	10.0	19.0	14.3 (2.6)	8.0	19.0	0.59
LOS (days)	20.7 (11.2)	5.0	48.0	13.5 (8.37)	3.0	41.0	<0.01
%EBW – Admit	78.7 (12.7)	61.5	118.0	81.25 (9.8)	58.8	103.0	0.34
%EBW – Discharge	85.12 (11.2)	71.2	120.0	85.5 (8.4)	60.2	104.0	0.76
Change in %EBW	6.77 (4.93)	0.44	17.2	4.98 (4.1)	−4.8	16.4	0.10
Calories – Admit (Kcals)	1185 (65.4)	1000	1300	1781 (315.2)	1500	3000	
Calories – Discharge (Kcals)	3019 (897.0)	1400	4500	3150 (784.8)	1800	5500	0.52
Change in Calories (Kcals)	1833 (906.8)	200	3300	1369 (725.3)	0	3200	0.02
	Frequency (%)			Frequency (%)			*P*-value
Female	81.0			84.9			0.67
Diagnosis:							0.20
Anorexia-restrict	47.6			72.7			
Anorexia-binge/purge	23.8			13.6			
Other Specified Eating Disorder	9.5			4.6			
ARFID	19.1			9.1			
Vegetarian history	14.3			13.6			0.94
Vegan history	9.5			7.6			0.78
Previously overweight/obese	23.8			18.2			0.57
First time admission	81.0			87.9			0.42
Insurance							0.02
Private	57.1			81.8			
Public	42.9			18.2			
Race/Ethnicity:							0.34
Caucasian	76.2			66.7			
Latino	9.5			13.6			
African-American	4.8			0			
Asian	4.8			10.6			
Mixed/Other	4.7			9.1			
Family history:							
Eating Disorder	14.3			39.4			0.03
Anxiety	23.8			28.8			0.66
Depression	47.6			48.5			0.94
Obsessive-Compulsive disorder	4.8			10.6			0.42
Drug/Alcohol abuse	38.1			34.9			0.79
NG/NJ Tube	28.6			9.1			0.02

*%EBW* % of Expected Body Weight, *ARFID* Avoidant Restrictive Food Intake Disorder, *NG/NJ* Nasogastric/Nasojejeunal

**Table 2:** Vital signs and Laboratory values of patients admitted to the Medical Behavioral Unit (*n* = 87)

	Low Calorie (*n* = 21)			High Calorie (*n* = 66)			
	Mean (S.D.)	Min	Max	Mean (S.D.)	Min	Max	*P*-value
Temperature	36.4 (0.8)	35.0	39.0	36.2 (0.5)	35.0	37.0	0.17
Orthostatic Vital Signs:							
Lying Systolic Blood Pressure	107 (12)	89	135	104 (12)	83	137	0.24
Lying Diastolic Blood Pressure	67 (11)	49	88	61 (9)	80	63	0.03
Lying Heart Rate (bpm)	64 (24.5)	40	121	57 (17)	31	116	0.18
Standing Systolic Blood Pressure	104 (13.3)	88	131	101 (11)	72	128	0.45
Standing Diastolic Blood Pressure	67 (9.5)	49	83	64 (12)	40	103	0.88
Standing Heart Rate (bpm)	71 (25.5)	39	125	73 (21)	42	113	0.75
Labs:							
Sodium (mmol/L)	140.4 (3.8)	134.0	148.0	141.1 (3.3)	131.0	147.0	0.20
Potassium (mmol/L)	3.8 (0.9)	3.0	7.0	3.6 (0.6)	3.0	5.0	0.27
Chloride (mmol/L)	102.7 (4.3)	95.0	113.0	102.0 (2.9)	92.0	111.0	0.80
Bicarb (mmol/L)	26.6 (3.6)	18.0	32.0	27.5 (2.6)	20.0	33.0	0.23
BUN (mg/dl)	13.2 (7.2)	2.0	31.0	15.8 (6.9)	1.0	37.0	0.14
Creatinine (mg/dL)	0.1 (0.03)	0.00	1.0	0.03 (0.17)	0.0	1.0	0.21
Glucose (mg/dL	87.1 (30.4)	54.0	180.0	83.0 (17.1)	31.0	142.0	0.45
Phosphorus (mg/dL)	3.7 (0.6)	2.0	4.0	3.8 (0.7)	1.0	5.0	0.72
Magnesium (mg/dL)	1.7 (0.5)	1.0	2.0	1.89 (0.3)	1.0	2.0	0.07
AST (U/L)	34.1 (13.4)	10.2	76.0	35.4 (12.0)	10.0	74.0	0.69
ALT (U/L)	38.3 (15.5)	15.0	66.0	31.9 (12.3)	14.0	98.0	0.05
Prealbumin (mg/dL)	19.1 (6.2)	10.0	30.0	20.9 (4.2)	11.0	32.0	0.15
Vitamin D (ng/mL)	35.6 (7.7)	23.0	44.0	37.8 (13.5)	15.0	79.0	0.66
WBC (thousand/uL)	5.8 (1.6)	3.5	11.0	5.4 (1.4)	1.6	9.7	0.26
Hgb (g/dL)	12.7 (1.2)	11.0	14.0	12.9 (1.3)	8.7	16.0	0.54
CRP (mg/dL)	0.7 (0.4)	0.5	2.2	0.60 (0.26)	0.5	1.9	0.30
Estradiol (pg/mL)	7.8 (7.7)	2.0	24.0	13.4 (15.3)	1.0	65.0	0.17
Testosterone (ng/mL)	8.0 (5.3)	2.0	12.0	24.5 (23.2)	1.4	74.0	0.25
ECG:							
QTc (ms)	0.410 (.031)	0.346	0.457	0.408 (.026)	0.335	0.470	0.81
Cardiac Monitoring:							
Lowest HR overnight – Admit (bpm)	49.4 (14.1)	29.0	78.0	43.8 (10.1)	31.0	79.0	0.05
Lowest HR overnight – Discharge (bpm)	68.8 (18.7)	45.0	105.0	54.7 (9.5)	42.0	79.0	<0.001
Change in HR	18.9 (16.0)	−10.0	49.0	10.5 (7.6)	−21.0	31.0	<0.01
	Frequency (%)			Frequency (%)			
Hypophosphatemia^a^	57.1			51.5			0.65
Hypomagnesemia^a^	52.4			51.5			0.94
Hypokalemia^a^	19.1			4.6			0.03

*bpm* beats per minute, *HR* Heart Rate

^a^Frequency of the development of low electrolyte levels in the first 72 h of admission

In the multivariable analysis (Table 3), starting on a higher calorie diet was associated with a lower change in HR (−6.3 bpm, S.D. 2.9, *p* = 0.03), controlling for initial %EBW, NG/NJ tube placement, and other demographic characteristics. There was no effect on absolute change in %EBW or rate of change in %EBW. While starting on a higher calorie diet was associated with lower change in calories during the admission (−526.5, S.D. 208.6, *p* = 0.01), it was not associated with the rate of change in calories. Length of stay was associated with NG/NJ tube placement (7.2 days, S.D. 2.7, *p* = 0.009) and inversely associated with %EBW on admission (−0.4, S.D. 0.1, *p* < 0.001). However, if children who were on NG/NJ tube feeds were removed from the analysis (*n* = 12), length of stay was significantly associated with starting on a higher calorie diet (−4.67 days, S.D. 2.20, *p* = 0.04) (full model not shown in table).

**Table 3:** Relationship between starting on a high calorie nutritional rehabilitation protocol and inpatient outcomes: Change in %EBW, Change in Calories, Rate of Change in %EBW, Rate of Change in Calories, Length of Stay, Change in Weight, and Change in Heart Rate

	Change in %EBW		Change in Calories (kcals)		Rate of Change in %EBW		Rate of Change in Calories (kcals)	
	Estimate (S.E.)	*p*-value	Estimate (S.E.)	*p*-value	Estimate (S.E.)	*p*-value	Estimate (S.E.)	*p*-value
Higher Calorie Refeeding Diet	−1.07 (1.04)	0.31	−526.5 (208.6)	0.01	0.01 (0.08)	0.88	−13.2 (12.4)	0.29
%EBW on admission	−0.22 (0.04)	<0.0001	−12.6 (8.9)	0.16	−0.01 (0.00)	0.03	1.7 (0.5)	0.002
Age	−0.14 (0.20)	0.49	86.8 (39.9)	0.03	−0.00 (0.02)	0.99	3.6 (2.4)	0.13
Race/Ethnicity								
Caucasian (reference)	0.0		0.0		0.0		0.0	
Latino	1.19 (1.36)	0.38	−47.2 (272.3)	0.86	0.11 (0.10)	0.29	4.2 (16.2)	0.80
African American	−5.68 (4.08)	0.17	−1352.1 (815.1)	0.10	−0.28 (0.32)	0.38	−55.3 (48.6)	0.26
Asian	3.02 (1.43)	0.04	−19.7 (287.3)	0.94	0.36 (0.11)	0.002	−23.2 (17.1)	0.18
Mixed/Other	1.23 (1.47)	0.41	154.2 (294.8)	0.60	0.14 (0.11)	0.22	−12.3 (17.6)	0.49
Insurance Status (Public)	2.28 (1.03)	0.03	174.6 (207.0)	0.40	0.10 (0.08)	0.23	−26.8 (12.3)	0.03
Diagnosis								
Anorexia-restrict (reference)	0.0		0.0		0.0		0.0	
Anorexia-binge/purge	−0.26 (1.27)	0.84	−277.8 (254.9)	0.28	−0.09 (0.10)	0.43	−30.4 (15.2)	0.05
Other Specified Eating Disorder	0.67 (1.85)	0.72	−337.3 (370.8)	0.37	0.05 (0.14)	0.72	0.3 (22.1)	0.99
ARFID	−1.11 (1.38)	0.42	−198.0 (276.7)	0.48	−0.07 (0.11)	0.52	−36.9 (16.5)	0.03
NG/NJ tube	1.59 (1.20)	0.19	−171.2 (240.1)	0.48	0.01 (0.09)	0.89	−48.1 (14.3)	0.001
	Length of Stay (days)		Change in Weight (kg)			Change in Heart Rate (bpm)		
	Estimate (S.E.)	*p*-value	Estimate (S.E.)		*p*-value	Estimate (S.E.)		*p*-value
Higher Calorie Refeeding Diet	−3.2 (2.3)	0.18	−0.52 (0.51)		0.32	−6.3 (2.9)		0.03
%EBW on admission	−0.4 (0.1)	<0.001	−0.09 (0.02)		<0.0001	−0.4 (0.1)		0.001
Age	0.4 (0.4)	0.35	0.15 (0.10)		0.12	−0.3 (0.5)		0.53
Race/Ethnicity								
Caucasian (reference)	0.0		0.0			0.0		
Latino	−0.5 (3.1)	0.86	0.66 (0.67)		0.33	0.6 (3.8)		0.87
African American	6.3 (9.2)	0.50	−1.79 (2.01)		0.38	−1.4 (11.0)		0.90
Asian	0.6 (3.2)	0.85	0.49 (0.71)		0.49	−5.4 (3.9)		0.16
Mixed/Other	0.9 (3.3)	0.80	0.42 (0.73)		0.56	−1.1 (4.0)		0.78
Insurance Status (Public)	4.6 (2.3)	0.05	0.76 (0.51)		0.14	4.1 (2.8)		0.15
Diagnosis								
Anorexia-restrict (reference)	0.0		0.0			0.0		
Anorexia-binge/purge	1.2 (2.9)	0.67	−0.04 (0.63)		0.95	0.1 (3.5)		0.98
Other Specified Eating Disorder	−1.0 (4.2)	0.82	−0.32 (0.91)		0.72	1.5 (5.0)		0.76
ARFID	0.4 (3.1)	0.89	−0.54 (0.68)		0.43	1.0 (3.8)		0.80
NG/NJ tube	7.2 (2.7)	0.009	0.74 (0.59)		0.22	0.1 (3.3)		0.96

Multivariable linear regression models were created to examine the relationship between starting on a high calorie nutritional rehabilitation protocol and several outcomes. All models controlled for age of patient, % expected body weight on admission, race/ethnicity, insurance status, diagnosis, and whether an NG/NJ tube was used during the admission. Adjusted estimates with standard errors and *p*-values are shown for each model

Initial calorie level was not associated with the development of hypophosphatemia, hypomagnesemia, or hypokalemia on admission or in the first 72 h, controlling for rate of calorie change, age, race/ethnicity, insurance, diagnosis, and NG/NJ tube placement (Tables 4 and 5). Rate of calorie change was also not associated with electrolyte abnormalities. However, %EBW was associated with hypophosphatemia on admission and trended towards hypomagnesemia such that those with higher %EBW had lower odds of developing these electrolyte abnormalities.

**Table 4:** Factors associated with Electrolyte Abnormalities Associated with Refeeding Syndrome on admission

	Hypophosphatemia		Hypomagnesemia		Hypokalemia	
	OR (95% CI)	*p*-value	OR (95% CI)	*p*-value	OR (95% CI)	*p*-value
Higher Calorie Refeeding Diet	0.92 (0.24, 3.50)	0.91	0.89 (0.23, 3.43)	0.87	>100 (<0.01, >100)	0.38
%EBW on admit	0.94 (0.88, 1.00)	0.05	0.94 (0.88, 1.00)	0.06	0.04 (<0.01, 36.97)	0.36
Rate of change in calories	1.00 (0.99, 1.01)	0.51	1.00 (0.99, 1.01)	0.56	1.36 (0.73, 2.54)	0.33

Multivariable logistic regression models were used to determine whether level of nutritional rehabilitation on admission was associated with hypophosphatemia, hypomagnesemia, or hypokalemia on the first day of admission. All models controlled for % expected body weight on admission, rate of change in calories, as well as age race/ethnicity, insurance status, diagnosis, and NG/NJ tube status

**Table 5:** Factors associated with Electrolyte Abnormalities Associated with Refeeding Syndrome in the first 72 h of admission

	Hypophosphatemia		Hypomagnesemia		Hypokalemia	
	OR (95% CI)	*p*-value	OR (95% CI)	*p*-value	OR (95% CI)	*p*-value
Higher Calorie Refeeding Diet	0.79 (0.22, 2.89)	0.72	0.82 (0.22, 2.96)	0.76	0.16 (0.01, 2.44)	0.19
%EBW on admit	0.96 (0.90, 1.01)	0.14	0.96 (0.91, 1.02)	0.18	1.05 (0.94, 1.18)	0.39
Rate of change in calories	0.99 (0.98, 1.01)	0.12	0.99 (0.98, 1.01)	0.17	0.98 (0.94, 1.01)	0.12

Multivariable logistic regression models were used to determine whether level of nutritional rehabilitation on admission was associated with hypophosphatemia, hypomagnesemia, or hypokalemia within the first 72 h of admission. All models controlled for % expected body weight on admission, rate of change in calories, as well as age race/ethnicity, insurance status, diagnosis, and NG/NJ tube status

A secondary analysis was conducted on severely malnourished patients who were <75% EBW on admission (*n* = 26). Starting on a higher calorie level compared to the lower calorie level was not associated with increased risk of developing hypophosphatemia (69% vs 60%, *p* = 0.65), hypomagnesemia (63% vs. 50%, *p* = 0.53), or hypokalemia (6% vs 10%, *p* = 0.72) in the first 72 h. In other analyses, severely malnourished patients who were started on a lower calorie diet were more likely to have an NG/NJ tube than those who were started on a higher calorie diet (30% vs. 0%, *p* = 0.02). Additionally, they were more likely to have more than one admission to the unit than those severely malnourished patients who were started on a higher calorie diet (40% vs. 6%, *p* = 0.03).

## Discussion

This paper includes a heterogeneous sample of adolescents diagnosed with all DSM-5 restrictive eating disorders and included a subgroup of severely malnourished patients presenting at <75% EBW. The study aimed to determine any clinical sequelae associated with starting on a higher calorie diet than traditionally recommended. Our results support the emerging literature suggesting that higher calorie nutritional rehabilitation can be safe and effective [7–18].

Consistent with findings from previous studies [11–13], incidence of electrolyte abnormalities in our sample was not associated with the rate of caloric advancement or initial calorie level. Rather, initial hypophosphatemia was more common among patients with a lower %EBW on admission, suggesting that the degree of body depletion is a more important predictor of electrolyte abnormalities. Previous studies have suggested that patients presenting with a weight <80% of median body weight are at elevated risk for electrolyte abnormalities associated with refeeding syndrome [3, 5]. Our data suggested that with every 1% decrease in %EBW on admission, the odds of hypophosphatemia increased by 6%. However, among our subset of severely malnourished patients presenting at <75% EBW, starting at a higher calorie diet was not associated with a higher risk of hypophosphatemia, hypomagnesemia, or hypokalemia. This subgroup was relatively small to draw definitive conclusions about the safety of higher calorie diets in this low-weight group, and additional studies with larger sample sizes are needed.

Higher calorie diets (1781 kcals, range 1500–3000 kcals) had no effect on the rate of change in %EBW which is consistent with findings by Golden et al. [11]. Interestingly, higher calorie diets were not associated with shorter lengths of stay when NG/NJ tube fed patients were included in the analysis. Patients needing NG/NJ tubes typically had significant behavioral components to their food refusal that required more prolonged behavioral intervention. They also had medical complications (e.g., superior mesenteric artery syndrome, intractable vomiting) that delayed the start of safe oral feeding, thus increasing length of stay. Therefore, the use of NG/NJ tubes appears to reflect a more severe population that needed a longer length of stay to resolve these issues. However, in post-hoc analysis removing this sub-population who received NG/NJ feeds, higher calorie diets were again associated with shorter lengths of stay (−4.6 days, S.D. 2.2, *p* = 0.04) which is consistent with the previous research [11, 13].

It is also interesting to note that in our sample of patients with a broad spectrum of disease characteristics, only half had low serum phosphorus and magnesium levels. However, without supplementation, many in this group resolved their electrolyte abnormality simply with oral nutrition. This suggests that prophylactic supplementation against refeeding syndrome, even among patients receiving high calorie diets, may be unnecessary if refeeding labs are closely observed. While daily lab draws have their own potential consequences, close observation may be a viable option and could prevent the use of unnecessary medications [21].

While this study adds to the existing literature on nutritional rehabilitation by including a broader range of diagnoses and disease characteristics, there were some limitations. A larger sample size would have allowed for stratified analyses among different %EBW groups so we could more clearly determine the risk of refeeding syndrome among patients with differing degrees of malnutrition. Additionally, physician clinical judgment played a role in initial calorie determination and calorie advancement that was difficult to quantify. This may have affected the lack of association between electrolyte abnormalities and initial calorie level, since those with low electrolyte levels could have been systematically started on lower calorie diets. However, initial calorie levels were often determined before lab results were back, thereby limiting this potential confounder. Due to the nature of self-reported data, we were also unable to accurately measure the rate of recent weight loss, which likely affects cardiovascular stability and risk of refeeding syndrome; this should be included in future analyses. Finally, we can only make limited conclusions in a descriptive or observational study, and a prospective randomized control trial is necessary for more definitive conclusions regarding the safety of higher initial calorie prescriptions.

## Conclusion

The results of this study continue to support the use of higher calorie nutritional rehabilitation diets among a heterogeneous group of patients with all DSM5 restrictive eating disorders, including those who are severely malnourished. Low %EBW on admission appears to be a more important predictor of electrolyte abnormalities than initial calorie level or rate of caloric advancement. Larger studies are required to demonstrate the safety of higher calorie diets, particularly in severely malnourished patients.
